# Association between physical activity and burnout among healthcare providers in Saudi Arabia: A cross-sectional study

**DOI:** 10.1097/MD.0000000000051148

**Published:** 2026-10-16

**Authors:** Mshari Alghadier

**Affiliations:** a Department of Health and Rehabilitation Sciences, Prince Sattam bin Abdulaziz University, Al-Kharj, Saudi Arabia.

**Keywords:** burnout, Copenhagen Burnout Inventory (CBI), healthcare providers, International Physical Activity Questionnaire (IPAQ), physical activity, Saudi Arabia

## Abstract

Burnout among healthcare providers is a major occupational health concern, associated with reduced quality of care, medical errors, and workforce attrition. Physical activity (PA) has been proposed as a factor associated with lower burnout, yet evidence from Saudi Arabia remains limited. This cross-sectional study examined the association between PA and burnout among healthcare providers in Saudi Arabia. A total of 110 healthcare providers (physicians, pharmacists, nurses, physiotherapists, and occupational therapists) completed a self-administered survey. Sociodemographic and professional data were collected, PA was assessed using the International Physical Activity Questionnaire–Short Form (IPAQ-SF), and burnout was measured with the Copenhagen Burnout Inventory (CBI). PA was categorized as low, moderate, or high, and total and domain-specific CBI scores (personal, work-related, client-related) were calculated. One-way ANOVA examined differences in burnout across PA levels. Multiple linear regression and logistic regression were used to evaluate PA influence, adjusting for age, gender, and profession. Higher levels of PA were associated with lower burnout among healthcare providers in Saudi Arabia. Although causality cannot be inferred from this cross-sectional design, given the limitations of convenience sampling and unmeasured occupational confounders, the findings suggest that PA may be one of several modifiable factors worth considering in occupational health strategies aimed at reducing burnout in Saudi healthcare settings. Most participants reported moderate (50.9%) or low (41.8%) burnout; and 7.3% reported high burnout level. High PA was common (57.3%), followed by moderate (26.4%) and low (16.4%) PA. Burnout differed significantly across PA levels for total and all domain scores (all *P* < .001; η^2^ = 0.23–0.34), with the high-PA group showing the lowest burnout. In linear regression, compared with high PA, low PA was associated with a 24.55-point higher total CBI score (*B* = 24.55, 95% CI: [17.05, 32.05], *P* < .001) and moderate PA with a 14.81-point higher score (*B* = 14.81, 95% CI: [8.61, 20.99], *P* < .001), while age, gender, and profession were not significant. In logistic regression, each additional 1000 MET-min/wk of PA was associated with a 50% reduction in the odds of moderate/high burnout (adjusted OR = 0.50, 95% CI: [0.39–0.65], *P* < .001), independent of covariates.

## 1. Introduction

Burnout among healthcare providers is increasingly recognized as a critical occupational health issue. It is commonly characterized by physical and emotional exhaustion, reduced energy, and feelings of ineffectiveness, which can undermine professional performance and quality of care.^[1-3]^ Globally, burnout prevalence estimates vary widely, ranging from approximately one-quarter to more than half of healthcare providers, depending on the setting, profession, definition, and measurement tool used.^[4,5]^ High workload, emotional demands of patient care, staff shortages, administrative burdens, and inadequate organizational support are important contributors to burnout.^[6]^ Persistent burnout has been associated with increased medical errors, reduced patient satisfaction, absenteeism, and increase staff turnover,^[7,8]^ highlighting the importance of addressing it to protect healthcare providers and maintain patient safety and quality of care.^[9]^

Physical activity (PA) has been proposed as a potentially modifiable factor associated with burnout. Regular PA is linked to improved mood, reduced stress, better sleep, and enhanced physical health, which may reduce vulnerability to emotional exhaustion and occupational strain.^[10]^ Consistent engagement in PA may also promote resilience in high-stress occupational environments such as healthcare.^[11]^ Within the Job Demands–Resources model, PA may function as a personal resource by supporting coping, recovery, and psychological detachment from work-related stressors.^[12,13]^ In addition, PA may support mood regulation, stress management, self-efficacy, and resilience, all of which have been associated with better psychological well-being and lower burnout^[10,14-17]^

Evidence from observational studies in different countries suggests that healthcare providers who engage in higher level of PA report lower burnout, although findings are not entirely consistent and may be influenced by occupational, cultural, and methodological factors.^[12,18]^ Systematic reviews have similarly linked higher PA with lower exhaustion and suggested that activity-based interventions may reduce burnout-related exhaustion.^[10,14]^ Comparable association have been reported in medical and university students, although the optimal PA dose remains uncertain.^[19]^ In the Middle East, a large survey of healthcare providers conducted during the COVID-19 pandemic found lower burnout scores among participants who engaged in PA than among inactive participants.^[20]^ In contrast, a study of nurses in Qatar found no significant association between PA level and burnout, although moderate PA was associated with greater compassion satisfaction.^[21]^

In Saudi Arabia, evidence regarding the association between PA and burnout remains limited and heterogeneous. A study on respiratory therapists found no significant association between PA and burnout, however, the study was conducted during the COVID-19 pandemic, which may have influenced both activity patterns and burnout levels.^[22]^ In contrast, studies have reported that lower exercise frequency was associated with higher burnout among medical students suggesting a possible relationship between PA and burnout in this population.^[23,24]^ Although these studies provide important local evidence, they focused on single professional or student populations and used different measurement approaches. Consequently, it remains unclear whether PA is associated with burnout among a multidisciplinary healthcare workforce assessed using validated tools.

The rapid expansion of the Saudi healthcare system, increasing service demands, and ongoing health-sector reforms have placed additional pressures on healthcare providers.^[25-28]^ Long working hours, shift work, staffing limitations, and high patient caseload may further increase occupational stress and burnout risk. Understanding potentially modifiable factors associated with burnout is therefore important for supporting workforce well-being and informing individual- and organizational-level strategies. Despite the recognized burden of burnout and growing interest in PA in Saudi Arabia, evidence remains limited regarding this association across diverse healthcare professions and routine healthcare settings using clearly defined and standardized measures.^[29]^ Such evidence may contribute to national health priorities, including Saudi Vision 2030 and the Health Sector Transformation Program, which emphasize workforce well-being and increased PA in the population.^[30,31]^

Therefore, this study aimed to describe burnout level and PA among healthcare providers in Saudi Arabia, examine differences in burnout scores across PA categories, and assess whether PA was associated with burnout severity after adjusting for demographic and professional characteristics. It was hypothesized that higher PA levels would be associated with lower burnout and that this association would remain significant after adjustment for relevant demographic and professional characteristics.

## 2. Materials and methods

### 2.1. Study design and participants

This study employed a cross-sectional, questionnaire-based design and was reported in line with the Strengthening the Reporting of Observational Studies in Epidemiology (STROBE) recommendations.^[32]^ Healthcare providers working across Ministry of Health (MoH) clinical settings in Saudi Arabia (including hospitals and ambulatory care facilities) were invited to participate. Recruitment followed a non-probability, voluntary response (convenience) approach, with invitations disseminated through MoH institutional channels and professional WhatsApp groups. The invitation included brief study information and a link to the online questionnaire. Because dissemination occurred via broad email channels and message forwarding within WhatsApp groups, the number of individuals who received or viewed the invitation could not be determined and, therefore, a response rate could not be calculated. Given the size of the MoH workforce (~5,00,000), the final sample (n = 110) represents a small and potentially non-representative subset; accordingly, selection bias is possible (e.g., differential participation by workload, wellbeing, or interest in PA), and findings may not generalize to all healthcare providers in Saudi Arabia. Eligible participants included physicians, pharmacists, nurses, physiotherapists, and occupational therapists currently practicing in Saudi Arabia. Inclusion criteria were licensure as a healthcare provider, age ≥21 years, and ability to read and complete the Arabic-language survey. Participation was voluntary and uncompensated. Overall, 110 healthcare providers completed the questionnaire and were included in the analysis. No formal a priori sample size calculation was conducted; the sample was recruited on a feasibility basis. Post hoc power analysis were conducted retrospectively and indicated adequate power to detect medium-to-large effects (*f*^2^ ≥ 0.15) at conventional α levels (0.05); these post hoc estimates should be interpreted with caution, as they do not substitute for a priori sample size planning. Missing data were minimal across all measures and no imputation was required.

### 2.2. Data collection tools

Data were collected using a structured self-administered questionnaire comprising 3 main components. The first component captured sociodemographic and professional characteristics, including gender, age, marital status, nationality, profession, years of clinical experience, highest educational qualification, smoking status, and the presence of any chronic disease. The second component assessed PA using the International Physical Activity Questionnaire-Short Form (IPAQ-SF), which is a widely used, reliable and valid self-report instrument that captures PA.^[33]^ This instrument records the frequency (d/wk) and duration (min/d) of walking, moderate-intensity activity, and vigorous-intensity activity over the previous 7 days, as well as time spent sitting. Prior evaluations of Arabic IPAQ instruments (short and long forms) indicate generally acceptable psychometric performance in Arabic-speaking adult populations, including Lebanese cohorts^[34]^ and samples from Saudi Arabia and the Gulf region. Across these studies, Arabic IPAQ self-reports have shown satisfactory test–retest reliability and reasonable agreement with objectively measured PA (e.g., accelerometers or activity monitors).^[35-37]^ IPAQ-SF responses were scored according to the established IPAQ protocol and converted to total metabolic equivalent (MET) min/wk. Total weekly PA (MET-min/wk) was calculated by summing activity-specific MET-minutes across domains, where each domain was computed as minutes per day × days per week × the corresponding MET value (walking = 3.3 METs; moderate-intensity activity = 4.0 METs; vigorous-intensity activity = 8.0 METs). Participants were then categorized as having low, moderate, or high PA based on standard IPAQ cut-points. In selected analyses, PA category was also modeled as an ordinal variable (low = 1, moderate = 2, high = 3) to reflect increasing activity levels.

The final component measured burnout using the Copenhagen Burnout Inventory (CBI), which was developed as a generic tool to measure burnout in both healthcare and non-healthcare occupations.^[38]^ The CBI has demonstrated good reliability and validity across diverse settings and professions and offers clear scoring and classification thresholds, making it suitable for epidemiological and occupational studies.^[39]^ Arabic versions of the CBI have demonstrated acceptable psychometric properties in Arabic-speaking healthcare populations, including evidence of internal consistency and construct validity supporting the 3-domain structure (personal, work-related, and client-related burnout).^[40,41]^ It consists of 19 items divided into 3 subscales: personal burnout (6 items), work-related burnout (7 items), and client-related burnout (6 items). Items are rated on a Likert-type scale, and raw scores are converted to a 0 to 100 scale, with higher scores indicating greater burnout. Subscale scores are calculated as the means of the items within each domain, and an overall burnout score is derived by averaging all items. Although the CBI was originally developed as 3 distinct subscales, psychometric studies in healthcare and non-healthcare populations have demonstrated high intercorrelations among the subscales and have supported the derivation and use of a global CBI total score.^[42–44]^ In line with published classifications, CBI scores were categorized into 4 levels: scores below 50 were considered low burnout, scores from 50 to 74 moderate burnout, scores from 75 to 99 high burnout, and a score of 100 was defined as severe burnout. In the present sample, participants were ultimately classified into low, moderate, or high burnout categories, as no cases met the threshold for severe burnout.

### 2.3. Data analysis

All analyses were performed using R version 4.0.3 (R Foundation for Statistical Computing, Vienna, Austria). Categorical variables are summarized as frequencies and percentages, and continuous variables as means and standard deviations. Burnout was examined both as a continuous outcome (total and domain-specific CBI scores) and, for selected analyses, as a categorical outcome. Physical activity was operationalized in 2 ways to address complementary aims. First, IPAQ-SF categories (low/moderate/high) were used to provide clinically interpretable group comparisons. Second, continuous MET-min/wk were used to assess whether a dose–response association was evident and to generate predicted probability plots. To avoid multicollinearity, categorical and continuous PA variables were evaluated in separate models rather than entered simultaneously. Extreme PA values were screened and handled according to the IPAQ scoring protocol.

To compare burnout across PA levels, 1-way analysis of variance (ANOVA) was used with IPAQ category (low, moderate, high) as the independent variable and total and domain-specific CBI scores as dependent variables. Prior to ANOVA, Levene’s test for homogeneity of variance was conducted. Where the assumption was met (personal burnout: F(2107) = 0.476, *P* = .622), standard 1-way ANOVA with Tukey’s Honestly Significant Difference (HSD) post hoc test was used for pairwise comparisons. For the 3 outcomes where Levene’s test indicated a significant violation of variance homogeneity: work-related burnout (*F*(2107) = 4.854, *P* = .010), client-related burnout (*F*(2107) = 5.051, *P* = .008), and total CBI (*F*(2107) = 8.701, *P* = .0003), Welch’s ANOVA was used as the primary analysis, with Games–Howell post hoc tests for pairwise comparisons, given its robustness to heteroscedasticity and unequal group sizes (n = 18, 29, 63). Associations between PA category and continuous burnout were further examined using multivariable linear regression with total CBI score as the dependent variable; IPAQ category was entered as the primary exposure (high PA as the reference), with age, gender, and profession included as covariates. Model fit was summarized using the coefficient of determination (*R*^2^) and the overall *F*-test.

In addition, burnout was dichotomized as low versus moderate/high according to established CBI cutoffs, and associations with continuous PA were examined using binary logistic regression. Physical activity was modeled as MET 1000 (MET-min/wk divided by 1000), and odds ratios were interpreted per 1000 MET-min/wk to improve interpretability. To support model stability given the sample size, adjusted models were limited to age, gender, and profession. Model fit was evaluated using likelihood ratio tests (LRT) and pseudo-*R*^2^ indices, and adjusted odds ratios (OR) with 95% confidence intervals (CI) were reported. Statistical significance was set at *P* ≤ .05 (2-tailed). Prior to formal analyses, standard regression assumptions were verified. For linear regression, normality of residuals was assessed visually (*Q*-*Q* plots) and formally (Shapiro–Wilk test), homoscedasticity was evaluated via residuals versus fitted-value plots, and multicollinearity was examined using variance inflation factors (VIF < 5 for all predictors). For the logistic regression model, calibration was assessed using the Hosmer–Lemeshow test. No substantial violations of model assumptions were identified.

As an exploratory analysis, restricted cubic spline (RCS) functions were fitted within a logistic regression framework to assess a potential nonlinear association between continuous PA (MET-min/wk) and burnout outcomes. The RCS model was specified with 4 knots, placed at the fifth, 35th, 65th, and 95th percentiles of the MET-min/wk distribution. Binary burnout (CBI ≥ 50) was used as the outcome, and the median MET-min/wk was set as the reference value. A LRT formally tested for nonlinearity by comparing the 4-knot RCS model against a linear PA term, yielding χ^2^(2) = 10.11, *P* = .006, supporting the presence of a nonlinear association. Model-based adjusted predictions (and 95% CI) were generated across the observed PA range, holding age at its mean and gender and profession at reference categories (female; nurse), and visualized in Figure 2 to aid interpretation of potential threshold or diminishing-returns patterns. As a sensitivity analysis addressing sparse professional subgroups, physiotherapists and occupational therapists were combined into a single allied health category, and key regression models re-estimated; conclusions were consistent with the primary specification.

**Figure 1.**
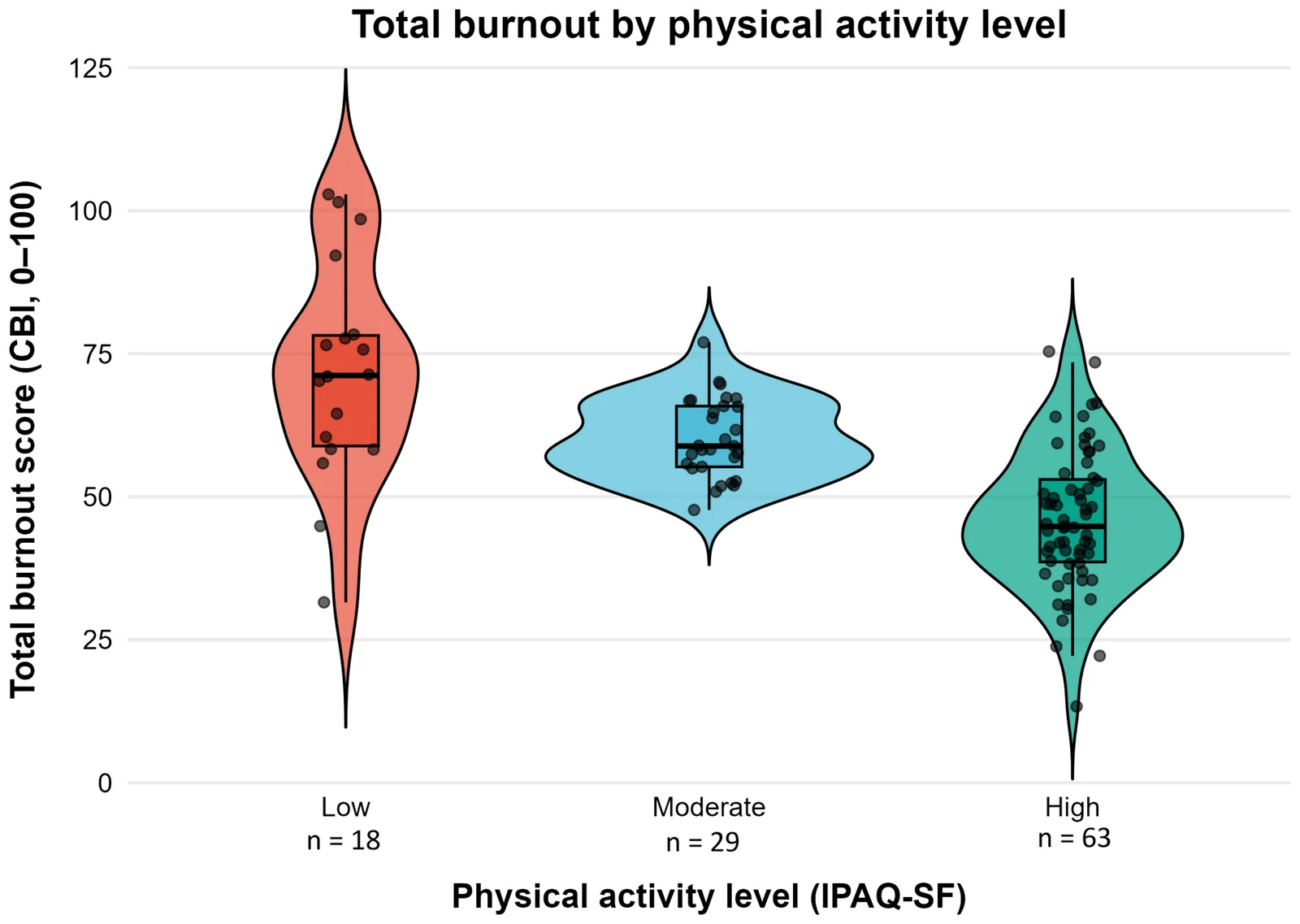
Distribution of total Copenhagen Burnout Inventory (CBI) scores by physical activity level. Violin plots with embedded boxplots and jittered data points show total burnout scores (0–100) across low, moderate, and high physical activity groups. The boxplot indicates the median and interquartile range, and individual points represent participants. Higher scores indicate greater burnout. CBI = Copenhagen Burnout Inventory.

**Figure 2.**
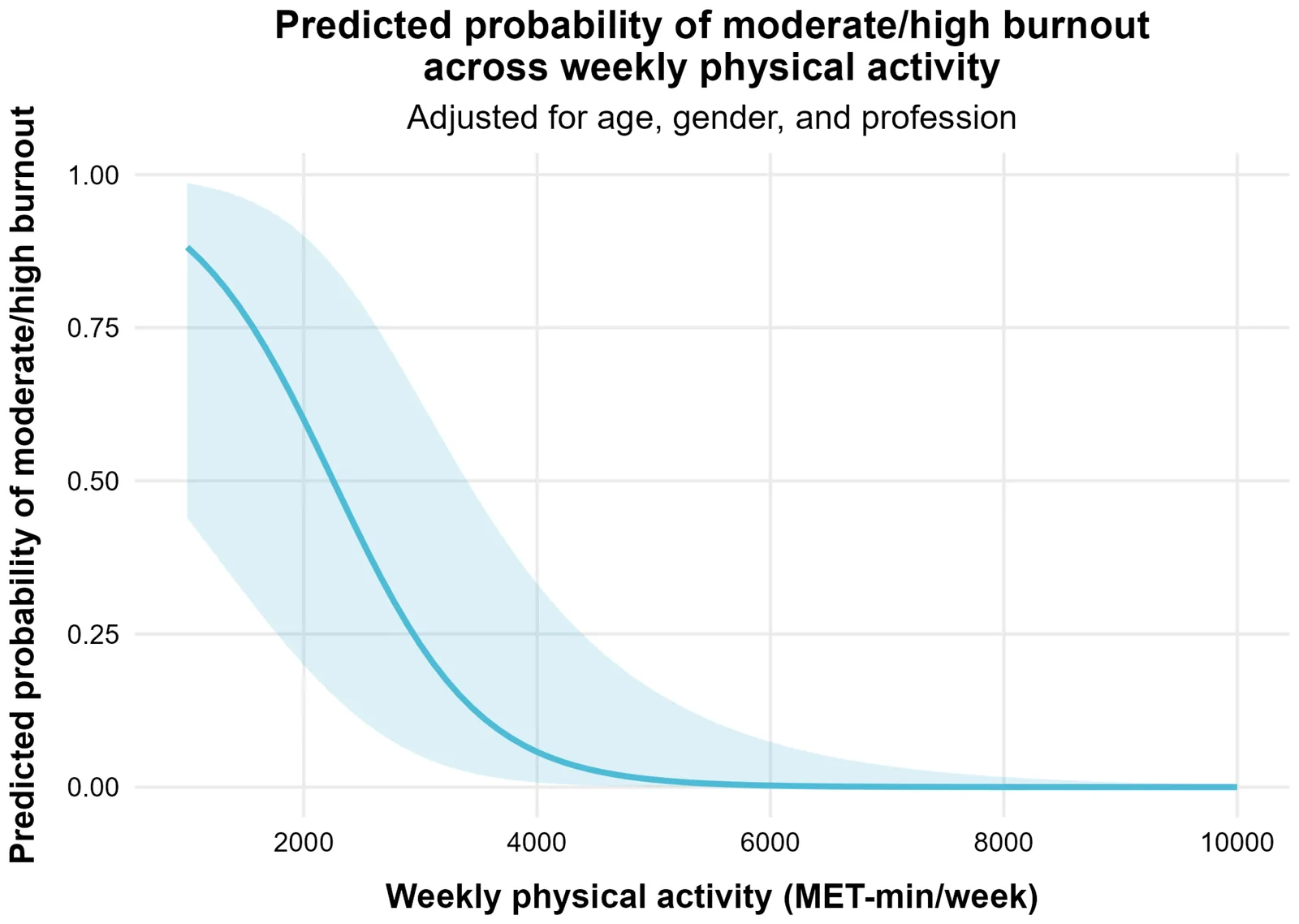
Predicted probability of moderate/high burnout across weekly physical activity (MET-min/wk) based on a restricted cubic spline (RCS) logistic regression model with 4 knots at the fifth, 35th, 65th, and 95th percentiles of MET-min/wk. The solid line represents the model-estimated probability of moderate/high burnout (CBI ≥ 50) across the observed PA range, referenced to the median MET-min/wk, holding age at its mean and gender and profession at their reference categories (female; nurse). The shaded band indicates the 95% confidence interval. A likelihood-ratio test comparing the 4-knot spline model against a linear term yielded χ^2^(2) = 10.11, *P* = .006. CBI = Copenhagen Burnout Inventory, IPAQ-SF = International Physical Activity Questionnaire–Short Form, MET = metabolic equivalent of task, RCS = restricted cubic spline.

### 2.4. Ethical considerations

Participation in the study was voluntary and anonymous. All participants provided informed consent electronically before completing the questionnaire. The study was conducted in accordance with the principles of the Declaration of Helsinki and received ethical approval from the Departmental Ethical Committee, Department of Health and Rehabilitation Sciences, Prince Sattam bin Abdulaziz University, Saudi Arabia (No. RHPT/025/007; date 15/05/2025).

## 3. Results

A total of 110 healthcare providers participated in the study (Table 1). The cohort was relatively young, with a near-balanced gender distribution, and consisted predominantly of Saudi, early- to mid-career clinicians. Most participants were married, reported at least 5 years of clinical experience, and held a bachelor’s degree, with fewer reporting postgraduate training. Physicians and pharmacists comprised the largest professional groups, alongside nurses and allied health professionals. Physical activity levels spanned low to high IPAQ categories, with high activity most prevalent.

**Table 1:** Demographic characteristics of the study sample (n = 110).

Variable	Category	Mean (SD)/ n(%)
Gender	Male	57 (51.8)
	Female	53 (48.2)
Age, yr, mean (SD)		32.2 (5.6)
Age group	21–25 yr	11 (10)
	26–30 yr	36 (32.7)
	31–35 yr	40 (36.4)
	36–40 yr	15 (13.6)
	41–45 yr	7 (6.4)
	Above 45 yr	1 (0.9)
Marital status	Married	57 (51.8)
	Single	53 (48.2)
Nationality	Saudi	104 (94.5)
	Non-Saudi	6 (5.5)
Years of experience	<2 yr	17 (15.5)
	2–5 yr	21 (19.1)
	5–10 yr	41 (37.3)
	10–15 yr	26 (23.6)
	>15 yr	5 (4.5)
Education level	Bachelor degree	85 (77.3)
	Master degree	25 (22.7)
Profession	Physician	32 (29.1)
	Pharmacist	29 (26.4)
	Nurse	20 (18.2)
	Physiotherapist	18 (16.4)
	Occupational therapist	11 (10)
Do you smoke?	Yes	22 (20)
	No	88 (80)
Do you suffer from chronic disease?	Yes	16 (14.5)
	No	94 (85.5)
IPAQ-SF	Low	18 (16.4)
	Moderate	29 (26.4)
	High	63 (57.3)
CBI total score category	Low	46 (41.8)
	Moderate	56 (50.9)
	High	8 (7.3)
CBI total score, mean (SD)		53.1 (16.5)
Personal burnout score, mean (SD)		55.8 (16.6)
Work-related burnout score, mean (SD)		53.3 (19.4)
Client-related burnout score, mean (SD)		50.3 (22.5)

Data presented as number (percentage) and mean (SD).

CBI = Copenhagen Burnout Inventory, IPAQ-SF = International Physical Activity Questionnaire-Short Form, SD = standard deviation.

One-way between-groups analyses of variance were conducted to examine differences in burnout across 3 PA levels (low, moderate, high) based on the IPAQ categories. Prior to ANOVA, Levene’s test confirmed homogeneity of variance for personal burnout *F*(2107) = 0.476, *P* = .622, but indicated heteroscedasticity for work-related burnout *F*(2107) = 4.854, *P* = .010, client-related burnout [*F*(2107) = 5.051, *P* = .008], and total CBI *F*(2107) = 8.701, *P* = .0003. There was a significant effect of PA on total burnout, personal burnout, work-related burnout, and client-related burnout across all domains. post hoc pairwise comparison confirmed that the high-PA groups had significantly lower burnout scores than both the low-PA and moderate-PA groups across all CBI domains (all adjusted *P* ≤ .001). For personal burnout, where variance homogeneity was confirmed, Tukey HSD tests were applied; the difference between the low-PA and moderate-PA groups was also significant (adjusted *P* = .035). For work-related burnout, client-related burnout, and total CBI score, where Levene’s test indicated heteroscedasticity, Games–Howell post hoc tests were used; the difference between the low-PA and moderate-PA groups was not significant for any of these domains (work-related: *P* = .277; client-related: *P* = .526; total CBI: *P* = .100). Table 2 presents the mean and standard deviations of total burnout score, personal burnout score, work-related burnout score, and client-related burnout score. It also presents the sensitivity analysis confirmed by Welch’s ANOVA and Games–Howell post hoc tests to the 3 CBI domains that violated variance homogeneity.

**Table 2:** The Copenhagen Burnout Inventory total score and personal, client-related, and work-related burnout scores of the sample, divided by physical activity levels.

Burnout domain	Low PA M (SD)	Moderate PA% M (SD)	High PA% M (SD)	*F*	η^2^	Levene’s *P*
Personal burnout	71.5 (12.9)^a^	60.6 (12.8)^b^	49.0 (15.5)^c^	19.30*	0.265	.622
Work burnout	68.8 (22.8)^a^	61.0 (13.6)^a^	45.4 (16.7)^b^	15.69*,†	0.243	.010
Client burnout	66.4 (26.4)^a^	59.9 (15.7)^a^	41.3 (19.8)^b^	15.12*,†	0.226	.008
Total CBI	68.9 (19.0)^a^	60.5 (7.6)^a^	45.2 (13.8)^b^	27.76*,†	0.338	<.001

PA levels classified using IPAQ criteria (low, moderate, high); n = 18 (Low), 29 (Moderate), 63 (High). Means within the same row sharing a superscript letter do not differ significantly. Tukey HSD post hoc applied for personal burnout (Levene *P* = .622; homogeneity assumption met). Games–Howell post hoc applied for work burnout, client burnout, and total CBI (Levene P < .05; homogeneity violated). *F* statistics for personal burnout are from 1-way ANOVA, df = (2, 107). *F* statistics marked † are from Welch’s ANOVA: work burnout df = (2, 39.93), client burnout df = (2, 40.21), Total CBI df = (2, 40.83).

ANOVA = analysis of variance, CBI = Copenhagen Burnout Inventory, IPAQ-SF = International Physical Activity Questionnaire-Short Form, M = mean, PA = physical activity, SD = standard deviation, η^2^ = eta-squared.

* *P* < .001.

† Welch’s ANOVA and Games–Howell post hoc applied (Levene *P* < .05).

The distribution of total burnout scores across PA categories is illustrated in Figure 1.

Multiple linear regression was used to examine whether PA, age, gender, and profession (physiotherapist and occupational therapist were merged into allied health category) associated with total burnout (CBI total score). The overall model was statistically significant, F(7, 102) = 8.18, *P* < .001, and explained 36% of the variance in burnout (*R*^2^ = 0.36, adjusted *R*^2^ = 0.31). Using high PA as the reference category, low PA was associated with a 24.55-point higher total CBI, and moderate PA with a 14.81-point higher score (Table 3).

**Table 3:** Multiple linear regression examining factors associated with total Copenhagen Burnout Inventory (n = 110).

Predictor	*B*	SE *B*	β	95% CI for *B*	*t*	*P*
Intercept	48.27	9.05		[30.31, 66.20]	5.33	<.001
Physical activity (ref: High)						
Moderate	14.81	3.12	0.39	[8.61, 20.99]	4.74	<.001
Low	24.55	3.78	0.55	[17.05, 32.05]	6.49	<.001
Gender (ref: Female)						
Male	−2.04	2.76	−0.06	[−7.51, 3.43]	−0.74	.462
Profession (ref: Nurse)						
Allied health	−1.63	4.09	−0.04	[−9.75, 6.49]	−0.39	.691
Pharmacist	−2.15	4.01	−0.05	[−10.12, 5.80]	−0.53	.592
Physician	−5.38	4.14	−0.14	[−13.59, 2.83]	−1.29	.197
Age (yr)	−0.15	0.24	−0.05	[−0.64, 0.34]	−0.60	.549

Dependent variable is the total Copenhagen Burnout Inventory (CBI) score (0–100), with higher scores indicating greater burnout. Physical activity was coded as a categorical predictor with high physical activity as the reference group; gender was coded with female as the reference group; profession was coded with nurse as the reference group.

*B* = unstandardized regression coefficient, CBI = Copenhagen Burnout Inventory, CI = confidence interval, SE *B* = standard error of *B*, β = standardized regression coefficient.

To further evaluate the association between PA and burnout severity, logistic regression was conducted with burnout dichotomized as low versus moderate/high. The adjusted model, including PA (MET-min/wk), age, gender, and profession (physiotherapist and occupational therapist were merged into an allied health category), was statistically significant (likelihood ratio χ^2^ = 47.3, *P* < .001; pseudo *R*^2^ = 0.34), with adequate model calibration confirmed by the Hosmer-Lemeshow test using g = 8 groups (recommended for n ≈ 100 to ensure adequate cells per group): χ^2^(6) = 11.94, *P* = .063. Physical activity, expressed per 1000 MET-min/wk, was significantly associated with lower burnout odds. Each additional 1000 MET-min/wk was associated with a 50% reduction in the odds of being in the moderate/high burnout group (*P* < .001). Neither age, gender, nor professional group was significantly associated with burnout status (all *P* > .05). In sensitivity analyses addressing sparse professional subgroups in the logistic models, physiotherapists and occupational therapists were combined into an allied health category, and PA was modeled as MET 1000 (MET-min/week ÷ 1000); results were directionally consistent and of similar magnitude to the primary model (Table 4).

**Table 4:** Logistic regression examining factors associated with moderate/high burnout (n = 110).

Predictor	*B*	SE *B*	OR	95% CI for OR	*z*	*P*
Intercept	5.41	1.69	223.63	[8.12, 6158.25]	3.19	<.001
Gender (ref: Female)						
Male	0.19	0.52	1.22	[0.43, 3.43]	0.37	.704
Profession (ref: Nurse)						
Allied health	−0.61	0.76	0.53	[0.11, 2.43]	−0.80	.421
Pharmacist	−1.06	0.77	0.34	[0.07, 1.57]	−1.37	.170
Physician	−0.45	0.81	0.63	[0.12, 3.15]	−0.55	.581
Physical activity (MET, per 1000 MET-min/wk)	−0.68	0.13	0.50	[0.39, 0.65]	−5.22	<.001
Age (yr)	−0.05	0.04	0.94	[0.85, 1.03]	−1.20	.227

Dependent variable is burnout status, coded 0 = low burnout and 1 = moderate/high burnout based on CBI cutoffs. Physical activity was entered as weekly MET-minutes divided by 1000; odds ratios, therefore, represent the change in odds of moderate/high burnout for each additional 1000 MET-min/wk. Gender was coded with female as the reference group; profession was coded with nurse as the reference group.

*B* = unstandardized logistic regression coefficient, CBI = Copenhagen Burnout Inventory, CI = confidence interval, MET = metabolic equivalent of task, OR = odds ratio, SE *B* = standard error of *B*.

To visualize the nonlinear association by the RCS analysis, model-based predicted probabilities of moderate/high burnout (CBI ≥ 50) were plotted from the RCS logistic regression model across the observed PA range (MET-min/wk), holding age at its mean and gender and profession at the reference categories (female, nurse). Figure 2 demonstrates a possible nonlinear dose–response association: the predicted probability of moderate/high burnout declines sharply as MET-min/wk increases, with the steepest reduction occurring as activity increases from low to moderate levels. At higher MET-min/wk values, the curve continues to decrease but begins to plateau, indicating diminishing marginal reductions in burnout probability at very high activity levels. Thus, Figure 2 adds to the regression table by visualizing the shape and magnitude of the association across the continuous PA distribution, rather than relying solely on a single OR estimate.

## 4. Discussion

This study examined the association between PA and burnout among 110 healthcare providers in Saudi Arabia. More than half of the participants had moderate or high burnout, while the majority were classified as having high PA. Higher PA was associated with lower total, personal, work-related, and client-related burnout scores after adjustment for age, gender, and profession. In the logistic regression analysis, each additional 1000 MET-min/wk of PA was associated with lower odds of moderate or high burnout. These findings indicate a graded inverse association between PA and burnout but do not establish a causal relationship.

The burnout burden observed here is consistent with findings from Saudi Arabia and other Arab countries. A systematic review reported highly variable prevalence estimates but substantial emotional exhaustion and depersonalization among healthcare providers.^[45]^ Recent Saudi studies have reported burnout among postgraduate healthcare trainees in Riyadh, physiotherapists, and staff working in intensive care, emergency, and pharmacy settings.^[27,28,46-51]^ These findings suggest that the burnout burden observed in the present study is part of a broader pattern of occupational distress among healthcare providers in the region rather than an isolated finding.

Our findings also align with international evidence linking higher PA with lower burnout, particularly exhaustion-related outcomes.^[10,14]^ Among healthcare providers, occupational and interventional studies have further suggested that PA is associated with lower stress and better mental health outcomes.^[52]^ Cross-sectional studies among nurses and other healthcare providers have similarly reported inverse associations between PA and burnout or job strain.^[53–55]^ The present study extends this evidence by showing that higher weekly MET-minutes were associated with progressively lower odds of moderate or high burnout among Saudi healthcare providers, even after adjustment for key demographic and professional characteristics.

Differences in burnout prevalence across Middle Eastern studies should be interpreted in light of important methodological and contextual variation. Reported estimates have ranged from 23% among Saudi medical students^[23]^ to 52.8% of healthcare providers in the United Arab Emirates reporting moderate-to-high burnout in at least 1 domain.^[20]^ Direct comparisons are limited because studies have used different instruments and cut-points, including the Maslach Burnout Inventory (MBI)–Student Survey, the MBI–Human Services Survey, and the Professional Quality of Life Scale, and have examined different populations, ranging from single-profession cohorts to mixed healthcare providers.^[21,22]^ A study conducted during the COVID-19 pandemic found no significant association between PA and burnout despite reporting low PA and high burnout.^[56]^ Differences in workload, professional composition, measurement, study design, and timing may therefore influence the observed relationship. Physical activity may represent a relevant modifiable correlate of burnout, but its relationship is likely context-dependent and should be considered alongside organizational and system-level factors.

Several mechanisms may plausibly explain the observed association, although they were not directly assessed. Physical activity has been associated with better mental health, fewer depressive and anxiety symptoms, improved sleep, and enhanced cognitive performance and emotional regulation.^[52]^ These outcomes are relevant to burnout, which the World Health Organization describes as a syndrome arising from chronic workplace stress that has not been successfully managed and involving energy depletion, increased mental distance or cynicism, and reduced professional efficacy.^[57-60]^ Psychosocial and recovery-related processes, including self-efficacy, mastery, psychological detachment, and relaxation, may also contribute to this relationship.^[54]^ However, because these factors were not measured, they should be regarded as possible explanations derived from previous research rather than conclusions from the present study.

The use of the CBI was another strength of this study. The CBI assesses personal, work-related, and client-related burnout and emphasizes exhaustion, in contrast to the more commonly used Maslach Burnout Inventory.^[61,62]^ It’s conceptualization has been considered consistent with the International Classification of Disease–11th Revision (ICD-11) description of burnout as a work-related exhaustion syndrome.^[63]^ The observation that PA was inversely associated with all the CBI domains suggests that the relationship was not limited to a single aspect of burnout but was evident across general and occupation-specific dimensions of burnout. Nevertheless, these domain-level associations should be interpreted as correlational and should not be taken to indicate that PA directly reduces burnout.

The use of the IPAQ-SF to quantify activity is consistent with contemporary epidemiological research. Systematic reviews and validation studies have reported acceptable test–retest reliability and moderate concurrent validity of the IPAQ-SF compared with accelerometer-based measures across diverse adult populations, including cultural and linguistic adaptations such as Arabic-speaking and other populations.^[35,64-67]^ However, the IPAQ-SF may overestimate PA compared to device-based measures, which is important when interpreting absolute activity levels observed in this study. The relatively high proportion of participants classified as having high PA may therefore reflect, at least in part, over-reporting or measurement inflation. Misclassification across PA categories could have influenced the observed associations, particularly if reporting differed according to burnout status. Future studies should therefore consider combining self-report with objective monitoring where feasible.

This study has several strengths. It addresses an important occupational health issue in Saudi Arabia, where evidence regarding modifiable correlates of burnout remains limited despite growing documentation of high burnout levels among nurses, physicians, residents, and other healthcare providers.^[27,28,45,46,51,68]^ The use of validated instruments, inclusion of several professional groups, and application of both linear and logistic regression analyses provided a complementary perspective on the PA–burnout relationship. In addition, examining PA both categorically (low, moderate, high) and continuously (MET-min/wk) allowed us to evaluate whether the findings were consistent across different representations of PA. The predicted probabilities from the logistic model also helped illustrate the inverse patterns across the observed PA range.

Several limitations should be considered. First, the cross-sectional design prevents causal inference, and reverse causation or residual confounding cannot be excluded. Second, convenience sampling, the absence of an a priori power calculation, and the modest sample size limit generalizability and may have reduced the precision of some estimates. Sparse categories in the logistic regression model should therefore be interpreted cautiously. Third, both PA and burnout were self-reported, creating potential recall and social desirability biases, while IPAQ-SF responses may have overestimated actual activity. Finally, important occupational and psychosocial factors – including workload, working hours, shift patterns, staffing, sleep quality, medical specialty, hospital type, workplace support, moral distress, and exposure to workplace violence – were not measured and may have confounded the observed associations.^[45,63]^ Future studies should incorporate these variables to provide a more complete assessment of the individual, occupational, and organizational factors associated with burnout.

The findings may have practical and policy implications, although they should not be interpreted as evidence that individual-level PA alone can prevent or resolve burnout. Physical activity may be considered one component of a broader occupational-health strategy that also addresses workload, staffing, leadership, organizational support, and workplace culture. In Saudi Arabia, this perspective is consistent with national initiatives under Vision 2030, the Health Sector Transformation, and Quality of Life programs, which emphasize both population health promotion and workforce well-being. Institutional PA initiatives, such as protected activity breaks, accessible exercise opportunities, active commuting support, group-based activities, and culturally appropriate digital tools, could be evaluated as part of multicomponent workplace interventions rather than implemented as stand-alone solutions.

Future research in Saudi Arabia and the wider region should use longitudinal and interventional designs to clarify the temporal relationship between PA and burnout. Prospective cohort studies could examine potential mediators, including sleep, recovery experiences, psychological detachment, and perceived organizational support. Randomized or quasi-experimental studies are also needed to evaluate the effectiveness, acceptability, and cost-effectiveness of workplace PA interventions, including structured activity breaks, step-based programs, and combined PA and psychological skills interventions.^[10,52]^ Combining device-based PA assessment with the IPAQ-SF would improve measurement accuracy, while qualitative studies could provide greater insight into barriers and facilitators to PA among healthcare providers. Finally, future research should evaluate PA within multicomponent burnout prevention programs that address work design, leadership, staffing, and team culture, reflecting the view that burnout is a complex occupational phenomenon rather than a purely individual stress response.

## 5. Conclusion

In summary, this study found that higher PA levels were associated with lower burnout among healthcare providers in Saudi Arabia, extending the literature to a Middle Eastern context where empirical data remain relatively limited. Given the cross-sectional design, these findings reflect associations only and should not be interpreted as evidence of causation. Physical activity may represent one of several modifiable correlates of burnout but is unlikely to be sufficient on its own and should be integrated with organizational and system-level strategies targeting workload, staffing, scheduling, and supportive work environments. Future studies using longitudinal and interventional designs are needed to determine whether PA promotion can causally reduce burnout in this context.

## Author contributions

**Conceptualization:** Mshari Alghadier.

**Data curation:** Mshari Alghadier.

**Formal analysis:** Mshari Alghadier.

**Funding acquisition:** Mshari Alghadier.

**Investigation:** Mshari Alghadier.

**Methodology:** Mshari Alghadier.

**Project administration:** Mshari Alghadier.

**Resources:** Mshari Alghadier.

**Software:** Mshari Alghadier.

**Supervision:** Mshari Alghadier.

**Validation:** Mshari Alghadier.

**Visualization:** Mshari Alghadier.

**Writing – original draft:** Mshari Alghadier.

**Writing – review & editing:** Mshari Alghadier.
